# Precision Medicine in Dermatology: MEK Inhibition and MAPK Pathway Modulation for Congenital Melanocytic Nevi

**DOI:** 10.3390/biomedicines14081833

**Published:** 2026-08-14

**Authors:** Alyssa Forsyth, Ochuwa Precious Imokhai, Kayla Fure, Rafael do Valle, Reem Ayoub, Alejandra Sataray-Rodriguez, Gabriella Martinez, Amanda Brooks

**Affiliations:** 1 Texas College of Osteopathic Medicine, University of North Texas Health, Fort Worth, TX 76107, USA; 2 Montana College of Osteopathic Medicine, Rocky Vista University, Billings, MT 59106, USA; 3 Reno School of Medicine, University of Nevada, Reno, NV 89557, USA; alejandrasataray@gmail.com; 4 Medical College of Wisconsin, Milwaukee, WI 53226, USA; 5 Department of Research and Scholarly Activity, Rocky Vista University, Ivins, UT 80112, USA

**Keywords:** congenital melanocytic nevi, MAPK pathway, MEK inhibition, precision medicine, targeted therapy, pediatric dermatology

## Abstract

Congenital melanocytic nevi (CMN) are pigmented skin lesions present at birth with a variable risk of developing into melanoma over time. The mitogen-activated protein kinase signaling (MAPK) pathway plays a critical role in the development and progression of CMN, making it a key target for therapeutic interventions. Precision medicine aims to optimize therapeutic efficacy while minimizing adverse effects by tailoring treatments to individuals’ genetic and molecular profiles. This literature review examines the potential of precision medicine in managing CMN, with a specific focus on MEK inhibition within the MAPK pathway, summarizing current preclinical and clinical evidence, mechanisms of action, and therapeutic integration with surgical management strategies. Although early results are promising, the need for personalized treatment strategies, informed by genetic testing and biomarker profiling, is emphasized to improve the management of CMN and reduce the risk of malignant transformation. Further research is necessary to establish the long-term safety and effectiveness of MEK inhibition in CMN therapy, which could offer a more targeted and individualized approach to care.

## 1. Introduction

Congenital melanocytic nevi (CMN) are pigmented melanocyte nevi that are frequently considered moles and are often visible at birth or during the early weeks of life. Although all melanocytes are derived from neural crest cells, CMN are congenital because they arise from postzygotic proliferations of melanocytes within the dermis and dermoepidermal junction driven by activating mutations in the MAPK pathway. This distinguishes them from acquired nevi, which develop later in life due to different mutational events. CMN usually arise randomly, but there are some risk factors, such as having neurofibromatosis type 1 and a family history of CMN. CMN are present in about 1% of newborns. The physical appearance of CMN is characterized by dark-colored, oval, and smooth moles with distinct, demarcated borders. As patients grow, the CMN will likely grow with the child and reach its final size when growth stops near adulthood. CMN can become raised, verrucous, or papillomatous and may also become more pigmented as the child grows. The current significant complication of CMN is that they can predispose individuals to malignant melanoma [1].

Activating mutations in NRAS and BRAF result in constitutive, ligand-independent activation of the MAPK pathway, driving sustained melanocyte proliferation and contributing to the pathogenesis of CMN. NRAS encodes a protein in the RAS family of GTPases that regulates cell growth, while BRAF encodes a kinase that activates downstream MAPK signaling. NRAS and BRAF mutations are the most frequently observed drivers in CMN, with NRAS mutations present in up to 95% of large CMN and BRAF mutations more common in small to medium-sized CMN [2,3]. Once BRAF is activated, the MAPK signaling cascade leads to increased transcription of target genes. CMN persist throughout life and do not spontaneously involute. Malignant transformation occurs when additional genetic alterations accumulate, leading to uncontrolled proliferation and progression toward melanoma [4].

The MAPK pathway is essential in cell proliferation and transcription, thereby increasing tumor cell survival. Mutations in BRAF and NRAS are compelling targets for treating melanoma and other cancers. Trametinib was the first MEK inhibitor approved and has shown productive outcomes in treating cancers [5]. Additionally, blocking the pathway with MEK1/2 inhibitors is expected to be effective for patients with cancers with RAS/RAF dysfunctions [6].

Precision medicine relating to CMN focuses on tailoring therapy based on genetic drivers such as NRAS and BRAF mutations. By matching patients’ molecular alterations to targeted therapies such as MEK inhibitors, precision medicine can improve efficacy while minimizing unnecessary interventions. This review applies precision medicine concepts specifically to the management of CMN, with an emphasis on MAPK pathway inhibition and data-driven, individualized care [7].

The purpose of this review is to summarize the clinical features and risks of CMN, examine the genetic and molecular pathways underlying its development, and evaluate the potential of precision medicine—particularly MEK inhibition—as a management strategy. The review also highlights future directions for integrating targeted therapies with surgical and non-surgical care.

## 2. Literature Search Strategy

A narrative literature review was conducted using PubMed/MEDLINE, Scopus, and Google Scholar. Search terms included combinations of “congenital melanocytic nevi,” “MAPK pathway,” “MEK inhibition,” “NRAS mutation,” “BRAF mutation,” “trametinib,” “selumetinib,” “precision medicine,” “pediatric dermatology,” and “melanocytic proliferation.” No strict date restriction was imposed; however, preference was given to publications from 2010 onward to reflect contemporary clinical and molecular understanding. Articles were included if they addressed (1) the pathogenesis or molecular biology of CMN; (2) clinical management strategies, including surgical and laser approaches; (3) MEK or BRAF inhibitor use in CMN or related pediatric RASopathies; or (4) precision medicine frameworks applicable to CMN. Case reports, preclinical studies, clinical trials, systematic reviews, and meta-analyses were all considered eligible. Articles not available in English and those with insufficient methodological reporting were excluded.

## 3. Discussion

### 3.1. Congenital Melanocytic Nevi (CMN)

Congenital melanocytic nevi (CMN) are pigmented skin lesions present at birth, typically appearing as dark brown or black patches of varying sizes and shapes [8,9]. These nevi result from abnormal growth of melanocytes, which are the pigment-producing cells in the skin. CMN are relatively common and can present in many sizes, from small spots to large, extensive lesions. Although most CMN are benign, there is a variable risk that these lesions can transform into melanoma over time [10].

#### 3.1.1. Clinical Presentation, Classification, and Risk of Malignant Transformation

CMN are classified by projected adult size: small (<1.5 cm), medium (1.5–20 cm), large (20–40 cm), and giant (>40 cm) [8]. Smaller nevi typically carry minimal malignant risk, whereas large and giant CMN demonstrate a higher propensity for melanoma due to deeper dermal involvement and a greater melanocyte burden [10]. Clinical features may evolve with age, including darkening pigmentation, surface irregularity, papillomatous texture, and hypertrichosis. Risk factors for malignant transformation include lesion size, presence of satellite nevi, histologic depth, cellular atypia, and anatomic location, particularly the trunk and head/neck regions [11]. Ultraviolet exposure may further increase malignant risk.

#### 3.1.2. Associated Syndromes, Risk Factors, Genetic Drivers, and the Role of UV Radiation

Neurocutaneous melanosis (NCM) is a rare congenital syndrome characterized by the presence of large or multiple CMN in association with melanocytic proliferation within the central nervous system [1,12]. Patients with giant CMN or numerous satellite lesions are at the greatest risk for NCM and require careful neurologic surveillance, including early MRI of the brain and spine. Activating mutations in NRAS, BRAF, and KRAS drive constitutive activation of the MAPK pathway, leading to sustained melanocyte proliferation and increased malignant potential [13]. NRAS mutations are most frequently observed in large and giant CMN, whereas BRAF mutations predominate in smaller lesions.

Ultraviolet (UV) radiation represents an important environmental co-factor in the biology of CMN. UV-induced DNA damage can introduce additional somatic mutations in nevus cells that already harbor constitutively activating NRAS or BRAF alterations. UV exposure has been proposed as a second-hit event capable of destabilizing genomic integrity in congenital melanocytic nevus cells, potentially accelerating the accumulation of alterations required for malignant transformation [1,4]. The anatomic predilection of CMN for the trunk and head/neck regions is consistent with this model. Rigorous photoprotection with high-SPF broad-spectrum sunscreen, protective clothing, and UV-avoidance counseling beginning in infancy should be incorporated into all surveillance protocols and family education materials regarding CMN, regardless of lesion size or planned therapeutic approach.

Recent transcriptomic studies have revealed significant molecular heterogeneity within large and giant CMN. RNA-sequencing analyses demonstrate distinct gene expression profiles involving immune signaling, extracellular matrix remodeling, and metabolic pathways. Differential activation of MAPK, PI3K/AKT, and Wnt signaling cascades suggests biological diversity even among nevi harboring similar driver mutations. These findings highlight that CMN are not molecularly uniform lesions and support the need for individualized therapeutic strategies based on lesion-specific molecular signatures rather than mutation status alone.

Data on racial and ethnic differences in NRAS and BRAF mutation frequencies in CMN remain limited. Large multi-ethnic genomic studies are needed to clarify potential disparities and ensure equitable application of precision medicine.

### 3.2. Current Management Strategies for CMN

Management of CMN is individualized based on lesion size, location, risk profile, and psychosocial impact [14]. Strategies include active surveillance, surgical excision, or a combination of modalities. Dermoscopy and advanced imaging techniques play a critical role in precision medicine-based risk stratification by enabling early detection of malignant transformation and guiding individualized surveillance strategies.

#### 3.2.1. Indications for Active Surveillance

Active surveillance is typically indicated for small to medium-sized CMN that demonstrate stable morphology and lack clinical or dermoscopic signs of dysplasia or malignant transformation [10,15]. Conservative monitoring is particularly appropriate when surgical excision would result in significant cosmetic or functional compromise. In neonates and infants, longitudinal observation allows clinicians to track growth patterns and detect the emergence of satellite lesions. Parental education is an essential component, enabling early recognition of worrisome changes such as rapid growth, ulceration, or color variegation.

#### 3.2.2. Role of Dermoscopy and Imaging Techniques

Dermoscopy is a useful, non-invasive way to follow CMN over time. Lesions on the head, neck, or trunk often show a globular pattern, while nevi that develop later or on the limbs more often have a reticular pattern. Small dermal nests (≤1.5 mm) have been found incidentally in skin specimens, supporting the theory that some clinically late-onset lesions may in fact be subclinical congenital nevi [10]. For infants with large CMN accompanied by satellite lesions along the central back, screening for NCM is recommended [10]. Early gadolinium-enhanced MRI of the brain and spine, ideally within the first four to six months of life, improves detection because myelination at later stages can obscure melanotic deposits. Annual MRI follow-up is standard for asymptomatic patients with positive findings. Surgery is typically deferred for patients with active neurological symptoms until stability is confirmed [15].

#### 3.2.3. Excision Techniques and Reconstructive Considerations

Surgical excision remains the primary treatment for many CMN, particularly when there is concern over malignant transformation or when cosmetic improvement is desired [10]. The approach depends on lesion size, location, and patient age (Table 1). Small CMN are often removed in a single procedure with primary closure, whereas larger lesions may require staged removal using serial excision, tissue expansion, or skin grafting. Serial excision was completed in an average of three to four procedures performed several months apart, with very low complication rates [16].

#### 3.2.4. Limitations and Risks of Surgical Removal

Complete excision of large or giant CMN is often not feasible because of anatomic constraints and the likelihood of significant disfigurement [10,15]. Even after substantial resection, nevus cells can persist in deeper dermal or subcutaneous layers, meaning surgery cannot fully eliminate malignant potential [10]. Recent systematic reviews estimate the lifetime melanoma risk in the case of giant CMN to range between 0.7% and 2.9%, a span substantially lower than that noted in previous reports [17], supporting a shift toward individualized risk-based surveillance. Early intervention offers no proven oncologic advantage and introduces additional anesthetic risk in very young children [18]. Major wound-related complications occur in about 10% of surgical cases [19,20]. Laser therapies, while useful for cosmetic improvement, do not eliminate deeper nevus cells and are associated with high recurrence rates [21,22,23,24,25].

### 3.3. Overview of Mapk Pathway Therapies

**Figure 1:**
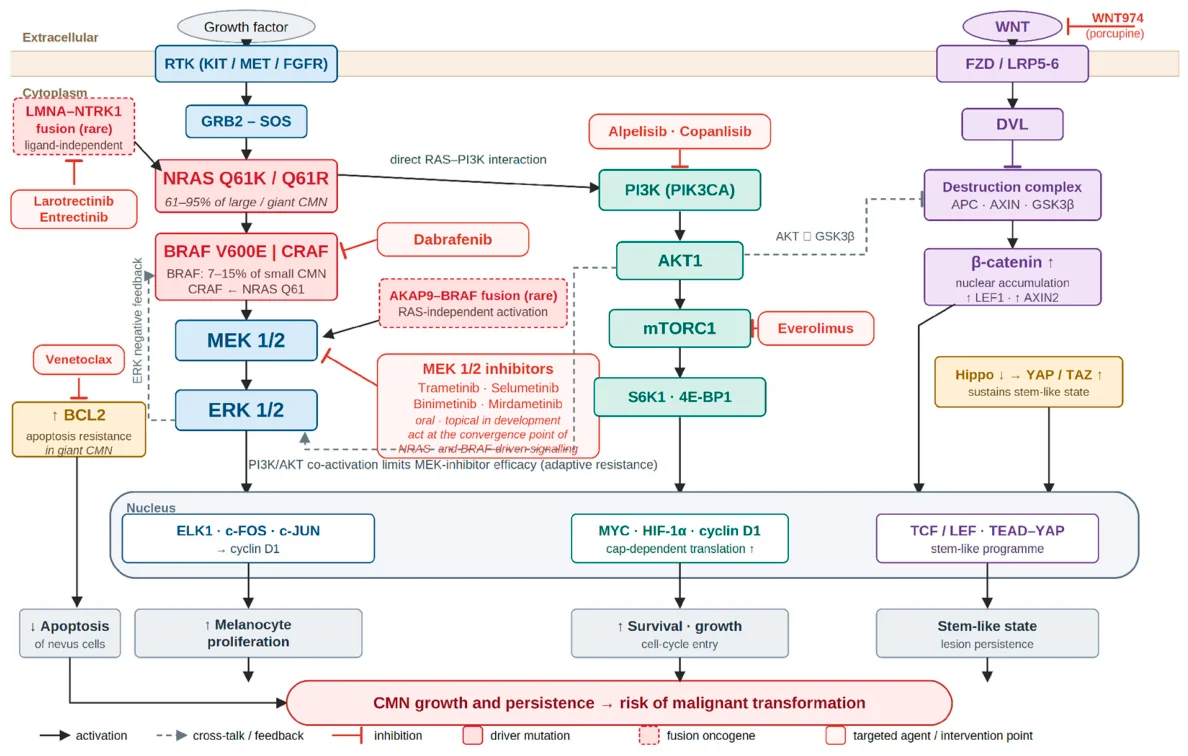
MAPK pathway activation, pathway cross-talk, and pharmacological intervention points in congenital melanocytic nevi (CMN). Canonical signaling (blue) proceeds from receptor tyrosine kinase (RTK) activation through GRB2–SOS, RAS, RAF, MEK1/2, and ERK1/2 to ELK1/c-FOS/c-JUN-driven cyclin D1 transcription. Oncogenic drivers reported in CMN are outlined in red: postzygotic NRAS Q61K/Q61R mutations (61–95% of large and giant lesions) generate a GTPase-deficient, GTP-locked protein that signals predominantly through CRAF; BRAF V600E predominates in small lesions (7–15%); and the rare fusion oncogenes AKAP9–BRAF and LMNA–NTRK1 (dashed outlines) confer constitutive RAS-independent or ligand-independent kinase activity. NRAS Q61 simultaneously activates PI3K/AKT/mTORC1 signaling (green) through direct RAS–PI3K interaction, so that AKT-dependent survival signaling and AKT-mediated inhibition of GSK3β persist during MEK blockade—a recognized mechanism of adaptive resistance that is compounded by loss of ERK-dependent negative feedback on RAF (dashed grey). Wnt/β-catenin signaling (purple) stabilizes β-catenin with upregulation of LEF1 and AXIN2, and reduced Hippo activity with YAP/TAZ accumulation cooperates with both axes to sustain a stem-like transcriptional program; BCL2 overexpression confers apoptosis resistance. Pharmacological intervention points are shown in red with bar-headed connectors: TRK inhibitors (Larotrectinib and entrectinib), BRAF inhibitors (dabrafenib), MEK1/2 inhibitors (trametinib, selumetinib, binimetinib, and mirdametinib—oral, with topical formulations in preclinical development), PI3K inhibitors (alpelisib and copanlisib), mTOR inhibitors (everolimus), porcupine inhibitors of WNT secretion (WNT974), and BCL2 inhibitors (venetoclax). MEK1/2 is highlighted as the convergence point of NRAS- and BRAF-driven signaling, providing the rationale for MEK inhibition across congenital melanocytic nevus mutation subtypes. Solid arrows denote activation, dashed arrows indicate cross-talk or feedback, and bar-headed lines denote pharmacological inhibition. APC = adenomatous polyposis coli; AXIN = axis inhibition protein; BCL2 = B-cell lymphoma 2; CMN = congenital melanocytic nevi; DVL = disheveled; ERK = extracellular-signal-regulated kinase; FZD = frizzled; GSK3β = glycogen synthase kinase 3 beta; LEF = lymphoid enhancer-binding factor; MEK = mitogen-activated protein kinase kinase; mTORC1 = mechanistic target of rapamycin complex 1; RTK = receptor tyrosine kinase; TCF = T-cell factor; TEAD = TEA domain transcription factor; YAP = Yes-associated protein.

Having reviewed current clinical management strategies, we now focus on molecular drivers and targeted therapeutic approaches. The MAPK signaling pathway (Figure 1) is essential in cell proliferation, differentiation, and survival. The pathway is initiated by growth factors binding to receptor tyrosine kinases, leading to activation of RAS and RAF. Downstream effects lead to activation of ERK, which regulates transcription factors that promote cellular proliferation and survival [26].

NRAS mutations, particularly at codon 61 (most commonly Q61K and Q61R substitutions), are commonly observed in CMN. These substitutions impair the intrinsic GTPase activity of NRAS, locking the protein in a constitutively GTP-bound, active conformation that drives persistent MAPK and PI3K/AKT signaling [2,3]. The mutations implicated in CMN are typically postzygotic, where a subset of cells carry the mutation, leading to a more localized presentation. In small and medium-sized CMN, approximately 70% of cases harbor NRAS mutations, whereas the remaining cases exhibit BRAF mutations. In larger CMN, NRAS mutations are found in approximately 94.7% of cases [2].

MEK inhibitors such as trametinib, cobimetinib, and binimetinib are particularly effective in treating mutant melanomas [27]. In a study by Pawlikowski et al., mice with NRAS mutations treated with the allosteric MEK inhibitor selumetinib showed significantly reduced melanocytic proliferation and suppressed congenital melanocytic nevus syndrome features [28]. CMN xenograft mice subjected to intradermal injections of binimetinib showed a decreased melanocyte population and proliferation [29]. Human trials are still required to establish full therapeutic potential.

#### 3.3.1. Rationale for Prioritizing the Mapk Pathway in CMN

The MAPK pathway is prioritized in CMN treatment due to its central role in disease pathogenesis. Postzygotic activating mutations in NRAS are identified in approximately 61–95% of cases of CMN, particularly in large and giant lesions, while BRAF mutations are more common in smaller nevi. These mutations act as driver events resulting in constitutive activation of the MAPK cascade and sustained melanocyte proliferation. Clinically approved targeted therapies already exist for this pathway, including MEK inhibitors such as trametinib and selumetinib [30,31], making MAPK modulation uniquely translatable to clinical practice [9]. Preclinical murine models and emerging case reports demonstrate that MEK inhibition suppresses nevus cell proliferation and improves clinical symptoms [28,29,30,32].

#### 3.3.2. Other Relevant Signaling Pathways

RNA-sequencing analyses of large and giant CMN have identified recurrent transcriptomic alterations beyond the MAPK axis. In the PI3K/AKT/mTOR pathway, upregulation of PIK3CA, AKT1, and downstream effector mTORC1 has been described in a subset of CMN, particularly those with concurrent NRAS mutations [33]. Critically, oncogenic NRAS Q61 mutations simultaneously drive the MAPK cascade and the PI3K/AKT axis via direct RAS-PI3K interaction, meaning that MEK inhibition alone may produce incomplete pathway suppression if AKT signaling remains active—a recognized mechanism of adaptive resistance in melanoma that is equally relevant to the biology of CMN [26,33].

In the Wnt/beta-catenin pathway, increased nuclear beta-catenin and upregulation of LEF1 and AXIN2 have been identified in transcriptomes of CMN, a finding consistent with a role for Wnt signaling in maintaining the nevus cell stem-cell-like state and resisting apoptosis [33,34]. The Hippo/YAP pathway cooperates with both MAPK and Wnt signaling to suppress differentiation and sustain proliferation in melanocytic neoplasia [33].

PI3K inhibitors (alpelisib and copanlisib) and mTOR inhibitors (everolimus) are under investigation in melanoma but have not yet entered clinical trials specifically for CMN [26]. Wnt pathway inhibitors including porcupine inhibitors (WNT974) are in early-phase oncology trials. These data argue that molecular profiling of CMN should extend beyond NRAS and BRAF to capture co-activating pathway alterations that may predict MEK inhibitor resistance and identify combination therapy candidates.

### 3.4. Precision Medicine and Mek Inhibition

In practice, the application of omics to the management of CMN follows a defined clinical workflow. At the time of biopsy or surgical excision, fresh-frozen or formalin-fixed paraffin-embedded (FFPE) lesional tissue is submitted for next-generation sequencing (NGS) panel testing, which simultaneously investigates NRAS, BRAF, KRAS, and co-occurring alterations in PI3K/AKT/mTOR and Wnt/beta-catenin pathway genes. RNA sequencing from the same specimen identifies fusion oncogenes—such as AKAP9-BRAF or LMNA-NTRK1—that would be missed by DNA-based panels alone and provides transcriptomic risk stratification [9,13]. Methylation arrays and epigenomic profiling can further distinguish CMN from melanoma at histologically ambiguous sites.

In dermatological clinics, mutation reports are reviewed in a multidisciplinary molecular tumor board involving dermatology, pediatric oncology, and clinical genetics to determine whether the driver mutation maps to an actionable target—for example, NRAS Q61 mutations directing consideration of MEK inhibition, or NTRK1 fusions directing consideration of TRK inhibitor therapy [35,36]. Surveillance frequency and imaging protocols are then calibrated to the molecular risk profile rather than lesion size alone. Gadolinium-enhanced MRI detects CNS melanocytic deposits associated with NCM [10,15]; PET imaging has theoretical utility for identifying metabolically active foci that may signal early malignant transformation; and CT may contribute to surgical planning for large or giant lesions. The convergence of lesion-specific molecular profiling with radiologic phenotyping supports truly individualized surveillance and treatment sequencing [37,38].

Although there are currently no published clinical trials examining the therapeutic value of MEK inhibition specifically in regard to CMN, several case reports illustrate promising results. In the case reported by Mir et al. (2019) [30], a 6-week-old girl harbored an AKAP9-BRAF fusion oncogene, confirmed by RNA sequencing and validated by fluorescence in situ hybridization. This fusion produces a truncated BRAF kinase lacking its regulatory RAS-binding and autoinhibitory domains, resulting in constitutive kinase activity functionally analogous to the BRAF V600E point mutation. BRAF-activating events signal directly to MEK1/2 without requiring upstream RAS activation, making MEK inhibition with trametinib a rational downstream intervention [13,27]. The patient was treated with oral trametinib and demonstrated a significant improvement in skin findings, with rapid improvement in pain and pruritus and no noted side effects [30].

In the cases reported by Copland et al. (2023) [32] and Kusters-Vandevelde et al. (2014) [39], NRAS mutation status was confirmed by Sanger sequencing or next-generation sequencing prior to treatment. NRAS Q61 mutations constitutively activate RAFs—predominantly CRAF—which phosphorylate MEK1/2, driving ERK-dependent transcription and melanocyte proliferation [3,26]. Trametinib, as an allosteric non-ATP-competitive inhibitor of MEK1 and MEK2, interrupts this signaling at the convergence point of both BRAF- and NRAS-driven activation, explaining why a clinical benefit is observed across distinct upstream mutations [9,28]. Both patients demonstrated clinically meaningful improvement—including lesion softening, reduction in nevus thickness, and decreased pain and pruritus—with manageable tolerability profiles.

#### 3.4.1. Pediatric Experience with Mek Inhibitors in Other Diseases

Pediatric-specific toxicity concerns for MEK inhibitors differ meaningfully from adult profiles and must be explicitly considered when extrapolating oncology data to CMN. Selumetinib is FDA-approved for children with neurofibromatosis type 1 (NF1) and inoperable plexiform neurofibromas, with clinical trials demonstrating significant tumor volume reduction and manageable toxicity [31]. Cardiac toxicity—specifically asymptomatic reductions in left ventricular ejection fraction—has been documented and requires baseline echocardiography with monitoring every 8–12 weeks [31,40]. In growing children, prolonged MEK/ERK suppression poses theoretical risks to skeletal development, given the established role of ERK signaling in chondrocyte proliferation and endochondral ossification. Ocular toxicity, including central serous retinopathy, requires ophthalmological surveillance adapted for pre-verbal infants. Endocrine surveillance including growth velocity monitoring should be incorporated into any pediatric trial protocol for CMN [31,40]. MEK inhibitors have also been explored in juvenile myelomonocytic leukemia and other RASopathies, providing valuable insight into pediatric dosing strategies and long-term developmental considerations [40].

#### 3.4.2. Emerging Targeted Therapies Beyond Mapk

Recent case reports have identified LMNA:NTRK1 fusion oncogenes in large CMN, expanding the molecular spectrum of actionable targets [35]. These fusions result in constitutive kinase activation, providing a rationale for TRK inhibitor therapy with agents such as larotrectinib and entrectinib, which are already approved for TRK-fusion-positive cancers [36]. Additionally, anti-BCL2 therapy has been reported to induce apoptosis in congenital melanocytic nevus cells via senolytic and immune-induction mechanisms [34]. These findings demonstrate that CMN may be amenable to multiple targeted strategies beyond MAPK inhibition, reinforcing the importance of comprehensive molecular profiling (Table 2).

#### 3.4.3. Genomic and Multi-Omics Approaches to Risk Stratification

Given that CMN arise from postzygotic somatic mutations, genomic profiling of lesional tissue represents a rational foundation for precision management. NGS of congenital melanocytic nevus tissue can confirm the driver mutation and simultaneously detect co-occurring alterations that may influence malignant potential or confer resistance to MEK-targeted therapy. Beyond single-gene testing, multi-omics integration—combining genomics, transcriptomics, proteomics, and epigenomics—may reveal lesion-specific molecular signatures that predict melanoma risk more accurately than lesion size alone [9].

Liquid biopsy—the detection and analysis of circulating cell-free DNA (cfDNA) or circulating tumor DNA (ctDNA) from peripheral blood or cerebrospinal fluid—represents a transformative opportunity for non-invasive longitudinal monitoring of CMN, particularly for patients with large or giant lesions for which repeated tissue sampling is impractical. Low-allele-frequency detection of NRAS Q61 or BRAF mutations in plasma cfDNA could serve as an early molecular signal of malignant transformation. In patients with NCM, CSF cfDNA sampling at the time of indicated lumbar puncture could detect CNS melanocytic clonal expansion. Incorporating prospective cfDNA collection into future registries and clinical trials regarding CMN would generate the longitudinal datasets needed to validate liquid biopsy as a surveillance tool for this population [9].

#### 3.4.4. Limitations and Ethical Considerations of Mek Inhibition in CMN

Age and developmental safety: CMN are most clinically significant in infancy and early childhood—the developmental window during which MAPK/ERK signaling plays fundamental roles in neural development, cardiac morphogenesis, and skeletal growth. Long-term developmental surveillance, including neurocognitive, cardiac, and endocrine assessments, must be incorporated into any future trial design.

Adverse events: Known toxicities include acneiform or maculopapular rashes, diarrhea, peripheral edema, elevated creatine phosphokinase, and reversible cardiac effects requiring echocardiographic monitoring [31,40]. Ocular toxicity warrants vigilance in pediatric use. Pyrexia and electrolyte disturbances are among the most common adverse events in pediatric combination MEK/BRAF inhibitor regimens [43].

Long-term follow-up: All published case reports on CMN involving MEK inhibitors have follow-up durations of weeks to months. Whether treatment produces durable histologic regression, prevents clonal evolution toward melanoma, or requires continuous dosing to maintain an effect is entirely unknown. Rebound proliferation following treatment discontinuation is a theoretical concern that warrants systematic study.

Cost and access: Trametinib and selumetinib are high-cost branded agents approved for specific oncologic indications. Their off-label use for CMN raises significant questions regarding payer access and cost-effectiveness. NGS panel testing and RNA sequencing remain inaccessible or unaffordable in most community dermatology settings and in the majority of low- and middle-income countries. Testing for a non-malignant indication is typically not covered by payers, and trametinib costs may range from tens of thousands to over one hundred thousand US dollars per patient per year when used in an off-label manner. Any precision medicine framework for CMN must be accompanied by a commitment to developing cost-effective diagnostic platforms and international registry infrastructure to support equitable access globally.

Ethical considerations: Administering systemic targeted therapy to neonates or infants for a largely benign congenital condition raises a distinct ethical threshold. The risk–benefit calculation must account for the revised lifetime melanoma risk estimate of 0.7–2.9% for giant CMN [17], the absence of proven oncologic benefits from early surgical excision [18], and the uncertainties regarding long-term MEK inhibitor safety in developing patients. Future clinical trials must incorporate robust ethical oversight, staged informed-consent processes as children mature to an age of assent, and long-term monitoring commitments clearly communicated to families.

### 3.5. Precision Medicine and Mek Inhibition: Tailoring Mapk Pathway Therapies to Improve the Management of CMN

Combination therapy with MEK and BRAF inhibitors, as opposed to BRAF inhibitor monotherapy, led to a decrease in BRAF-inhibitor-induced lesions such as squamous cell carcinoma but also an increase in pyrexia [41]. The combination of MEK and BRAF demonstrates the greatest efficacy in treating CMN that have progressed to melanoma, with improved overall survival and superior progression-free survival profiles [42]. Overall safety profiles of MEK/BRAF inhibitor combination therapy for pediatric patients support continued usage, with pyrexia and electrolyte disturbances being among the most common side effects [43]. Both the short-term and long-term efficacy of combination therapy support its consideration for both pediatric and adult patient populations.

Surgical resection presents challenges regarding large and giant CMN. Surgical resection of small CMN is highly efficacious but can result in scarring. For large CMN, skin grafting and numerous procedures may be required to achieve satisfactory removal and desirable cosmesis. Future studies must evaluate the potential efficacy of pharmacologic treatment in combination with surgical excision.

### 3.6. Future Directions

MEK inhibitors have shown promise in preclinical models of CMN, where they reduce melanocytic proliferation. However, their long-term value in human patients remains unproven. Large, prospective, multicenter trials are needed to evaluate clinical and histologic clearance, recurrence rates after discontinuation, cosmetic outcomes, and patient-reported quality of life. These studies should compare systemic and localized delivery to determine whether targeted application can minimize toxicity while maintaining efficacy. Until such data are available, MEK inhibitors should remain within investigational settings.

An emerging and particularly promising direction for congenital-melanocytic-nevus-directed MEK inhibition is the development of topical formulations—creams, gels, and nanoparticle-encapsulated carriers—designed to deliver therapeutic MEK inhibitor concentrations directly to the dermis while minimizing systemic absorption and associated toxicity. Topical MEK inhibitors including binimetinib-based gel formulations and trametinib in lipid nanoparticle vehicles have been explored in preclinical melanocytic models, with evidence of dermal penetration sufficient to suppress ERK phosphorylation in lesional cells [29]. Mirdametinib (PD-0325901) in a topical formulation has entered early clinical investigation for cutaneous neurofibromas in NF1, providing a relevant precedent for topical MAPK inhibition in a pediatric RASopathy [31]. For CMN—particularly large and giant lesions where systemic exposure is the primary safety concern in neonates—topical delivery represents a theoretically optimal strategy. Rigorous pharmacokinetic studies establishing skin penetration depth, systemic bioavailability, and optimal vehicle formulations for neonatal skin are needed before topical MEK inhibitors can enter congenital-melanocytic-nevus-specific clinical trials.

Genomic profiling offers an opportunity to move the management of CMN toward a precision medicine model. Sequencing can identify driver mutations that may predict responsiveness to targeted therapy and inform surveillance intensity. Standardized multi-omics protocols, embedded within prospective registries, are needed to translate biological insights into validated, clinically actionable biomarkers for risk stratification and therapy selection regarding CMN. The long-term goal is to shift from reactive treatment to proactive, risk-adapted care.

Combination strategies may ultimately define the next phase of therapy for CMN. MEK inhibition could be used to thin lesions before surgery, reducing the extent of excision and the complexity of reconstruction. Partial excision followed by laser treatment and adjuvant targeted therapy could achieve greater nevus cell clearance than any single modality. Immunotherapy, which has reshaped melanoma treatment, may have a role in preventing recurrence or eliminating residual cells after surgical intervention. Implementation will require coordination across dermatology, plastic surgery, pediatrics, and oncology. Long-term registry data and well-designed trials will be critical for refining these multimodal regimens.

## 4. Conclusions

CMN present complex clinical challenges due to their variable presentation, risk of malignant transformation, and impact on patient quality of life. Current treatment strategies center on risk-based surveillance and surgical excision. Recent advances in precision medicine have opened new possibilities for the management of CMN, particularly through MEK inhibition targeting the MAPK pathway. Early preclinical data and a small number of case reports suggest that MEK inhibitors may reduce nevus cell burden and improve clinical symptoms in NRAS- and BRAF-altered lesions; however, whether pharmacologic MAPK pathway inhibition translates to a reduction in the risk of malignant transformation remains unestablished and must be evaluated in prospective, controlled clinical trials before any such conclusion can be drawn. The integration of genetic profiling, multi-omics biomarker development, liquid biopsy, and topical drug delivery strategies will be instrumental in guiding individualized care. Continued research and innovation—alongside equitable access to genomic diagnostics and targeted therapies—are needed to establish evidence-based best practices that improve both clinical outcomes and patient experience in the management of CMN.

## Figures and Tables

**Table 1:** Management approach for congenital melanocytic nevi (CMN) by lesion size.

CMN Size	Typical Management	Role of Imaging	Surgical Options
Small (<1.5 cm)	Active surveillance; surgery if changes are detected	Not indicated	Single-stage excision
Medium (1.5–20 cm)	Active surveillance if stable; surgery if changes are detected	MRI if multiple lesions or concerning features are detected	Direct or serial excision
Large/Giant (>20 cm)	Surveillance for satellite lesions and NCM; surgery	Brain/spine MRI within 4–6 months of life	Staged excision (with reconstruction as needed)

**Table 2:** Molecular Drivers in CMN and Corresponding Targeted Therapeutic Strategies.

Molecular Alteration	Frequency	Pathway	Key Effectors	Inhibitor Class	Exemplar Agents	Status in CMN/Related Conditions	Route
NRAS Q61K/Q61R mutation	61–95% (large/giant CMN)	MAPK + PI3K/AKT	MEK, ERK, AKT, mTOR	MEK inhibitor	Trametinib, Selumetinib, Binimetinib	Case reports; preclinical models [28,29,30,32,39]	Oral systemic; topical investigational
BRAF V600E mutation	7–15% (small CMN)	MAPK	MEK, ERK	BRAF + MEK inhibitor	Dabrafenib + Trametinib	Case report [30]; approved in melanoma [41,42]	Oral systemic
AKAP9-BRAF fusion	Rare	MAPK (RAS-independent)	MEK, ERK	MEK inhibitor	Trametinib	Single case report [30]	Oral systemic
LMNA-NTRK1 fusion	Rare	TRK/RAS/MAPK	ERK, PI3K	TRK inhibitor	Larotrectinib, Entrectinib	Single case report [35]; approved in TRK-fusion cancers [36]	Oral systemic
PIK3CA/AKT1 co-activation	Subset; co-occurs with NRAS	PI3K/AKT/mTOR	AKT, mTOR, S6K	PI3K/mTOR inhibitor	Alpelisib, Everolimus	Early-phase trials in melanoma; not in CMN [33]	Oral systemic
Wnt/beta-catenin upregulation (LEF1, AXIN2)	Transcriptomic subset	Wnt/beta-catenin	beta-catenin, TCF/LEF	Porcupine inhibitor	WNT974	Early-phase oncology trials; not in CMN [33,34]	Oral systemic
BCL2 overexpression	Reported in giant CMN	Apoptosis resistance	BAX/BCL2 ratio	BCL2 inhibitor (senolytic)	Venetoclax	Preclinical/single case report [34]	Oral systemic

## Data Availability

No new data were created or analyzed in this study.

## Abbreviations

The following abbreviations are used in this manuscript:

BRAF	B-Raf Proto-Oncogene, Serine/Threonine Kinase
cfDNA	Cell-Free Deoxyribonucleic Acid
CMN	Congenital Melanocytic Nevi
CT	Computed Tomography
ctDNA	Circulating Tumor DNA
ERK	Extracellular-Signal-Regulated Kinase
FFPE	Formalin-Fixed Paraffin-Embedded
KRAS	Kirsten Rat Sarcoma Viral Oncogene
MAPK	Mitogen-Activated Protein Kinase
MEK	MAPK/ERK Kinase (Mitogen-Activated Protein Kinase Kinase)
MRI	Magnetic Resonance Imaging
NCM	Neurocutaneous Melanocytosis
NF1	Neurofibromatosis Type 1
NGS	Next-Generation Sequencing
NRAS	Neuroblastoma Rat Sarcoma Viral Oncogene
PET	Positron Emission Tomography
RAF	Raf Proto-Oncogene, Serine/Threonine Kinase
RAS	Rat Sarcoma Viral Oncogene Family
RTK	Receptor Tyrosine Kinase
UV	Ultraviolet

## References

[1] Moustafa D., Blundell A.R., Hawryluk E.B. (2020). Congenital melanocytic nevi. Curr. Opin. Pediatr..

[2] Charbel C., Fontaine R.H., Malouf G.G., Picard A., Kadlub N., El-Murr N., How-Kit A., Su X., Coulomb-L’Hermine A., Tost J. (2014). NRAS mutation is the sole recurrent somatic mutation in large congenital melanocytic nevi. J. Investig. Dermatol..

[3] Kinsler V.A., Thomas A.C., Ishida M., Bulstrode N.W., Loughlin S., Hing S., Chalker J., McKenzie K., Abu-Amero S., Slater O. (2013). Multiple congenital melanocytic nevi and neurocutaneous melanosis are caused by postzygotic mutations in codon 61 of NRAS. J. Investig. Dermatol..

[4] Roh M.R., Eliades P., Gupta S., Tsao H. (2015). Genetics of melanocytic nevi. Pigment. Cell Melanoma Res..

[5] Grimaldi A.M., Simeone E., Festino L., Vanella V., Strudel M., Ascierto P.A. (2017). MEK Inhibitors in the Treatment of Metastatic Melanoma and Solid Tumors. Am. J. Clin. Dermatol..

[6] Cheng Y., Tian H. (2017). Current Development Status of MEK Inhibitors. Molecules.

[7] Kosorok M.R., Laber E.B. (2019). Precision Medicine. Annu. Rev. Stat. Its Appl..

[8] Macneal P., Syed H.A., Patel B.C. (2025). Congenital melanocytic nevi. StatPearls [Internet].

[9] Tan S., Hu H., Xin X., Wu D. (2024). A clinical and biologic review of congenital melanocytic nevi. J. Dermatol..

[10] Price H.N., Schaffer J.V. (2010). Congenital melanocytic nevi-when to worry and how to treat: Facts and controversies. Clin. Dermatol..

[11] Kinsler V.A., O’Hare P., Bulstrode N., Calonje J.E., Chong W.K., Hargrave D., Jacques T., Lomas D., Sebire N.J., Slater O. (2017). Melanoma in congenital melanocytic naevi. Br. J. Dermatol..

[12] Martins da Silva V., Martinez-Barrios E., Tell-Marti G., Dabad M., Carrera C., Aguilera P., Brualla D., Esteve-Codina A., Vicente A., Puig S. (2019). Genetic Abnormalities in Large to Giant Congenital Nevi: Beyond NRAS Mutations. J. Investig. Dermatol..

[13] Roy S.F., Agim N.G., Mir A., Couts K.L., Vandergriff T., Robinson W.A., Bastian B.C., Frieden I., Yeh I. (2025). Congenital melanocytic nevi initiated by BRAF fusion oncogene with firmness, pruritus, and desmoplastic stroma. Br. J. Dermatol..

[14] Alikhan A., Ibrahimi O.A., Eisen D.B. (2012). Congenital melanocytic nevi: Where are we now? Part I. J. Am. Acad. Dermatol..

[15] Pastore L.M., Valentini R., Marghoob A.A. (2025). Congenital melanocytic nevi and risk of melanoma. Clin. Dermatol..

[16] Hassanein A.H., Rogers G.F., Greene A.K. (2015). Management of challenging congenital melanocytic nevi: Outcomes study of serial excision. J. Pediatr. Surg..

[17] Arad E., Zuker R.M. (2014). The shifting paradigm in the management of giant congenital melanocytic nevi: Review and clinical applications. Plast. Reconstr. Surg..

[18] Kinsler V., Bulstrode N. (2009). The role of surgery in the management of congenital melanocytic naevi in children: A perspective from Great Ormond Street Hospital. J. Plast. Reconstr. Aesthetic Surg..

[19] Gout H.A., Fledderus A.C., Lokhorst M.M., Pasmans S.G.M.A., Breugem C.C., Lapid O., van der Horst C.M.A.M. (2023). Safety and effectiveness of surgical excision of medium, large, and giant congenital melanocytic nevi: A systematic review and meta-analysis. J. Plast. Reconstr. Aesthetic Surg..

[20] Carmen Ceballos-Rodriguez M., Redondo P., Tomas-Velazquez A., Cieza-Diaz D., Carlos Lopez-Gutierrez J. (2021). Surgical outcomes and psychosocial impact of giant congenital melanocytic nevus surgery: A single-center case series of 136 patients. J. Pediatr. Surg..

[21] Al-Hadithy N., Al-Nakib K., Quaba A. (2012). Outcomes of 52 patients with congenital melanocytic naevi treated with UltraPulse Carbon Dioxide and Frequency Doubled Q-Switched Nd-Yag laser. J. Plast. Reconstr. Aesthetic Surg..

[22] August P.J., Ferguson J.E., Madan V. (2011). A study of the efficacy of carbon dioxide and pigment-specific lasers in the treatment of medium-sized congenital melanocytic naevi. Br. J. Dermatol..

[23] Lim J.M., Oh Y., Lee S.H., Cho M.Y., Chung K.Y., Roh M.R. (2019). Comparison of treatment options for small to medium congenital melanocytic nevi: A retrospective review of 119 cases. Lasers Surg. Med..

[24] Oh Y., Lee S.H., Lim J.M., Chung K.Y., Roh M.R. (2019). Long-term outcomes of laser treatment for congenital melanocytic nevi. J. Am. Acad. Dermatol..

[25] Eggen C.A.M., Lommerts J.E., van Zuuren E.J., Limpens J., Pasmans S.G.M.A., Wolkerstorfer A. (2018). Laser treatment of congenital melanocytic naevi: A systematic review. Br. J. Dermatol..

[26] Ullah R., Yin Q., Snell A.H., Wan L. (2022). RAF-MEK-ERK pathway in cancer evolution and treatment. Semin. Cancer Biol..

[27] McClure E., Carr M.J., Zager J.S. (2020). The MAP kinase signal transduction pathway: Promising therapeutic targets used in the treatment of melanoma. Expert Rev. Anticancer Ther..

[28] Pawlikowski J.S., Brock C., Chen S.C., Al-Olabi L., Nixon C., McGregor F., Paine S., Chanudet E., Lambie W., Holmes W.M. (2015). Acute Inhibition of MEK Suppresses Congenital Melanocytic Nevus Syndrome in a Murine Model Driven by Activated NRAS and Wnt Signaling. J. Invest. Dermatol..

[29] Rouille T., Aractingi S., Kadlub N., Fraitag S., How-Kit A., Daunay A., Hivelin M., Moguelet P., Picard A., Fontaine R.H. (2019). Local Inhibition of MEK/Akt Prevents Cellular Growth in Human Congenital Melanocytic Nevi. J. Invest. Dermatol..

[30] Mir A., Agim N.G., Kane A.A., Josephs S.C., Park J.Y., Ludwig K. (2019). Giant congenital melanocytic nevus treated with trametinib. Pediatrics.

[31] Gross A.M., Wolters P.L., Dombi E., Baldwin A., Whitcomb P., Fisher M.J., Weiss B., Kim A., Bornhorst M., Shah A.C. (2020). Selumetinib in Children with Inoperable Plexiform Neurofibromas. N. Engl. J. Med..

[32] Copland A., Sandaradura S.A., Wargon O. (2023). Congenital melanocytic naevus syndrome, is there a role for trametinib monotherapy?. J. Paediatr. Child Health.

[33] Luond F., Pirkl M., Hisano M., Prestigiacomo V., Kalathur R.K.R., Beerenwinkel N., Christofori G. (2021). Hierarchy of TGF-beta/SMAD, Hippo/YAP/TAZ, and Wnt/beta-catenin signaling in melanoma phenotype switching. Life Sci. Alliance.

[34] Yan J., Huang Y., Lu J., Chen J., Yang J., Zhang H., Zhao Y., Pi J., Liu X., Gu Y. (2025). Anti-BCL2 therapy eliminates giant congenital melanocytic nevus by senolytic and immune induction. Signal Transduct. Target. Ther..

[35] Nagarkar A., Turbeville J., Hinshaw M.A., Bennett D.D., Hagen J.W. (2025). Large Congenital Melanocytic Nevus With LMNA::NTRK1 Fusion: Expanding Targeted Therapy Options for Congenital Nevi and Melanoma. J. Cutan. Pathol..

[36] Hsiao S.J., Zehir A., Sireci A.N., Aisner D.L. (2019). Detection of Tumor NTRK Gene Fusions to Identify Patients Who May Benefit From Tyrosine Kinase (TRK) Inhibitor Therapy. J. Mol. Diagn..

[37] Dominiczak A.F., Padmanabhan S., Caulfield M., Sutherland K., Wang J., Jones J.K. (2023). Introducing Cambridge prisms: Precision medicine. Camb. Prism. Precis. Med..

[38] Lip S., Padmanabhan S. (2025). Introduction to precision medicine. Medicine.

[39] Kusters-Vandevelde H.V., Willemsen A.E., Groenen P.J., Kusters B., Lammens M., Wesseling P., Djafarihamedani M., Rabold K., Willemsen M.A., Vinke A.G. (2014). Experimental treatment of NRAS-mutated neurocutaneous melanocytosis with MEK162, a MEK-inhibitor. Acta Neuropathol. Commun..

[40] Dombi E., Baldwin A., Marcus L.J., Fisher M.J., Weiss B., Kim A., Whitcomb P., Martin S., Aschbacher-Smith L.E., Rizvi T.A. (2016). Activity of Selumetinib in Neurofibromatosis Type 1-Related Plexiform Neurofibromas. N. Engl. J. Med..

[41] Flaherty K.T., Infante J.R., Daud A., Gonzalez R., Kefford R.F., Sosman J., Hamid O., Schuchter L., Cebon J., Ibrahim N. (2012). Combined BRAF and MEK inhibition in melanoma with BRAF V600 mutations. N. Engl. J. Med..

[42] Grob J.J., Amonkar M.M., Karaszewska B., Schachter J., Dummer R., Mackiewicz A., Stroyakovskiy D., Drucis K., Grange F., Chiarion-Sileni V. (2015). Comparison of dabrafenib and trametinib combination therapy with vemurafenib monotherapy on health-related quality of life (COMBI-v). Lancet Oncol..

[43] Bouffet E., Geoerger B., Moertel C., Whitlock J.A., Aerts I., Hargrave D., Osterloh L., Tan E., Choi J., Russo M. (2023). Efficacy and Safety of Trametinib Monotherapy or in Combination With Dabrafenib in Pediatric BRAF V600-Mutant Low-Grade Glioma. J. Clin. Oncol..

